# A Modified NAR Scoring Model Incorporating Immune Infiltration Characteristics to Better Predict Long-Term Survival Following Neoadjuvant Radiotherapy in Rectal Cancer

**DOI:** 10.3390/life13112106

**Published:** 2023-10-24

**Authors:** Xueqing Zhang, Yibin Zeng, Hui Li, Qingyang Zhuang, Lirui Tang, Junxin Wu, Jinluan Li

**Affiliations:** Department of Radiation Oncology, Clinical Oncology School of Fujian Medical University, Fujian Cancer Hospital, Fuzhou 350014, China; xueqingzhang21@126.com (X.Z.); zyb9803@163.com (Y.Z.); lihui960815@gmail.com (H.L.); zqy199302@163.com (Q.Z.); tlr330306938@pku.edu.cn (L.T.)

**Keywords:** rectal cancer, neoadjuvant radiotherapy, prognosis, modified-NAR scoring model, immune infiltration

## Abstract

(1) Background: The neoadjuvant rectal (NAR) score has been developed as a prognostic tool for survival in locally advanced rectal cancer (LARC). However, the NAR score only incorporates weighted cT, ypT, and ypN categories. This long-term follow-up study aims to modify a novel prognostic scoring model and identify a short-term endpoint for survival. (2) Methods: The prognostic factors for overall survival (OS) were explored through univariate and multivariate analyses. Based on Cox regression modeling, nomogram plots were constructed. Area under the curve (AUC) and concordance indices were used to evaluate the performance of the nomogram. Receiver operating characteristic (ROC) analysis was conducted to compare the efficiency of the nomogram with other prognostic factors. (3) Results: After a long-term follow-up, the 5-year OS was 67.1%. The mean NAR score was 20.4 ± 16.3. Multivariate analysis indicated that CD8+ T-cell, lymphovascular invasion, and the NAR score were independent predictors of OS. The modified NAR scoring model, incorporating immune infiltration characteristics, exhibited a high C-index of 0.739 for 5-year OS, significantly outperforming any individual factor. Moreover, the predictive value of the nomogram was superior to the AJCC stage and pathological complete regression at 3-year, 5-year, and 10-year time points, respectively. Over time, the model’s predictions of long-term survival remained consistent and improved in accuracy. (4) Conclusions: The modified NAR scoring model, incorporating immune infiltration characteristics, demonstrates high accuracy and consistency in predicting OS.

## 1. Introduction

Colorectal cancer is the fourth most commonly diagnosed type of cancer and the second most common cause of cancer-related deaths in the world [1]. Neoadjuvant radiotherapy (nRT) and chemoradiotherapy followed by total mesorectal excision (TME) have become the standard treatment approach for locally advanced rectal cancer (LARC) [2,3]. However, the response to nRT is variable. It can range from pathological complete regression (pCR) to minimal effect or even tumor progression [4]. Additionally, assessing treatment efficacy typically relies on 3- or 5-year survival rates, which can be time-consuming and costly. Therefore, there is a need for early surrogate endpoints in oncology clinical trials to reduce patient numbers and trial duration [5]. Finding a new short-term endpoint for individual prognosis assessment and clinical decision making is essential.

Advancements in treatment have led to the development of various grading systems, including TNM staging, tumor regression grading (TRG), and pCR [6,7,8]. TNM staging system after nCRT is a key component in the clinical assessment of prognostic prediction and risk stratification, which helps to guide the clinical treatment decisions. Even though LARC patients with the same TNM staging receive similar treatment their outcomes vary [9]. The TRG system has been developed to evaluate tumor pathologic responses to neoadjuvant therapy [10]. However, there is still no consensus on whether TRG is a prognostic factor for LARC [11]. Only approximately 20% of patients achieved pathologic complete regression (pCR) and had favorable long-term survival [12]. This subset of patients have the opportunity to be treated with a “watch and wait” approach rather than radical surgery [13]. One such endpoint that has been proposed as a prognostic factor for rectal cancer is the neoadjuvant rectal (NAR) score. Valentini’s nomogram for overall survival is used to calculate the NAR score (OS) [14], which incorporates both clinical and pathological characteristics on the T stage and N stage before and after nRT [15]. Patients with low NAR scores have demonstrated favorable tumor outcomes [16] and have been evaluated in the Phase 2 Randomized Clinical Trial [17]. The previous study confirmed the predictive role of the NAR score for DFS and its surrogate at the individual patient level in a large randomized phase III trial. The NAR score may help oncologists to accelerate response-adapted therapeutic decisions. However, the German trial only showed advantages in terms of disease-free survival, not OS [16]. It remains unclear whether the NAR score offers better predictive accuracy for OS compared to other prognostic factors.

While immunotherapy has shown significant benefits in various cancer types, such as melanoma and lung cancer [18], these improvements were not seen in most rectal cancer patients [19]. Unlike other cancers, radiotherapy can exert an impact on the tumor microenvironment by eliciting systemic immune-mediated antitumor effects [20]. Until now, several clinical trials are underway to evaluate the role of neoadjuvant immunotherapy combined with radiotherapy in rectal cancer. Preliminary studies had shown that the combination of immunology and radiotherapy had led to promising results. It is believed that resistance to immunotherapy in microsatellite stable rectal cancer may be overcome by the potential immune-stimulating effect of radiation. Although most of the reported studies are small samples from phase 1–2 clinical trials, complete response (CR) rates have shown a good trend toward improvement [21]. A Chinese clinical trial of short-course radiotherapy (SCRT) combined with immunotherapy had shown a high pCR of 48% (13/27), which exceeded the results of the total neoadjuvant therapy model [22]. Studies to date have shown that lymphocyte levels are related to neoadjuvant response for cancer patients, including rectal, oesophageal, and breast cancer [23,24,25]. Emerging evidence suggests that high tumor-infiltrating lymphocytes (TIL) levels, particularly CD8+ T-cells, is related to improved prognosis for neoadjuvant rectal cancer patients [26]. A previous study has reported a correlation between higher NAR scores and various clinic-pathologic features, such as old age, positive cN status, and lower tumor differentiation [16]. However, a model incorporating immune cell infiltration characteristics to predict long-term prognosis for rectal cancer has not yet been developed.

Therefore, we aimed to modify the NAR scoring model by incorporating immune cell infiltration characteristics and applying it to LARC patients who received nRT, to establish a more efficient approach for promptly identifying the success or failure of experimental interventions and, thereby, improve clinical decision support.

## 2. Materials and Methods

### 2.1. Eligibility Criteria

In this retrospective study, we extracted and recorded detailed information from hospital records of LARC who underwent nRT with subsequent TME in our institution between February 2012 and September 2015. Criteria comprised histologically confirmed adenocarcinoma and staging Ⅱ (T3-4N0) or Ⅲ (TanyN+) rectal cancer based on the American Joint Committee on Cancer staging system, 8th Edition. Pelvic MRIs were conducted before and after nRT in all eligible patients. Patients diagnosed with metastatic disease or a Karnofsky performance score below 70 were excluded. All clinicopathological features and laboratory results were recorded. Finally, seventy-six cases were included in the study. Our study had the approval of the Ethics Committee of Fujian Cancer Hospital (NO. KT2022-120-01). Moreover, the Ethics Committee waived individual consent as the analysis involved retrospective examination of patient information, without utilizing any individual patient identifiable information.

### 2.2. Treatment and Follow-Up

Patients underwent either short-course radiotherapy (SCRT; 25 Gy in 5 fractions) or long-course chemoradiotherapy (LCRT; 50 Gy in 25 fractions) as neoadjuvant therapy. Concurrent chemotherapy during LCRT consisted of capecitabine (825 mg/m^2^) administered two times a day, 5 days a week. The treatment options are determined by the clinician. Typically, patients with high tumor burden are selected for long course radiotherapy. The primary cancer was included in the clinical target volume (CTV), as well as the anorectal, mesorectal, pre-sacral, and internal iliac lymph nodes. Intensity-modulated radiation therapy (IMRT) was used to deliver the RT regimens. All plans were created for a Varian Truebeam accelerator using 6 MV photon beam. After nRT, TME was performed in all cases, either one week after SCRT or 6 to 8 weeks after LCRT, by expert surgeons.

For the first 2 years, all patients were followed every 3 months, and then every 6 months for 3 years, and then on an annual basis thereafter.

### 2.3. NAR Scores

The NAR score compromised of weighted cT to take into account the downstaging of tumors, as well as ypT and ypN categories, which are affected by neoadjuvant therapy [15]. The NAR score equation is as follows: NAR = [5ypN − 3(cT − ypT) + 12]^2^/9.61, where cT in {1,2,3,4}, ypT in {0,1,2,3,4}, and pN in {0,1,2} [14]. Subsequently, the NAR scores encompass 24 different scores ranging from 0–100. The pCR is equal to 0 points, while 100 points represent progress.

### 2.4. TRG

Two pathologists independently evaluated H&E staining of surgical specimens after nRT. A five-grade Mandalay system (Fibrosis/Tumor Relationship Tumor Relationship) was used for TRG—TRG 1: complete regression, no viable cancer cells; TRG 2: rare residual cancer cells scattered through the fibrosis; TRG 3: increased number of residual cancer cells, fibrosis predominates; TRG 4: residual cancer outgrowing fibrosis; and TRG 5: no regression [27]. The patients were divided into two groups: those with complete regression (TRG 1) and those with incomplete regression (TRG 2–5).

### 2.5. Clinicopathological Variable Stratification

Receiver operating characteristic (ROC) analysis from the Cutoff Finder (http://molpath.charite.de/cutoff, accessed on 6 April 2023) [28], were employed to classify PD-1, CD3+ T-cell, and CD8+ T-cell as dichotomous variables, with optimal cutoff values of 5.5%, 12.5%, and 9.0%, respectively.

The variables in the cohort were categorized as follows: age at diagnosis (<60, ≥60), gender (male or female), body mass index (BMI, <18.5 kg/m^2^, 18.5–24.9 kg/m^2^, ≥25 kg/m^2^), distance to anal margin (<5 cm, ≥5 cm), lymphovascular invasion (yes or no), neural invasion (yes or no), tumor nodules (yes or no), mucinous adenocarcinoma (yes or no), pathologic differentiation (poorly, moderately), cT stage (cT2, T3, T4), cN stage (cN0, N1, N2), ypT stage (ypT0, ypT2, ypT3, ypT4), ypN stage (ypN0, ypN1, ypN2), PD-1 (<5.5%, ≥5.5%), CD3+ T-cell (<12.5%, ≥12.5%), CD8+ T-cell (<9.0%, ≥9.0%), pCR (yes, no), nRT (short course, long course), neoadjuvant chemotherapy (yes or no), adjuvant radiotherapy (yes or no), and adjuvant chemotherapy (yes or no).

### 2.6. Statistical Analyses

OS refers to the time from diagnosis to the time of death. Cox regression models were used for analysis of survival and identification of variables with prognostic significance. Statistical significance was defined as a *p* value less than 0.05. Subsequently, based on the multivariate analysis, a nomogram was constructed showing OS rates at 3, 5, and 10 years. The predictive ability was evaluated using Harrell’s C-index. Receiver operating characteristic (ROC) analysis was performed using the Delong test. The efficiency of the nomogram was compared with other factors.

SPSS version 22.0 (IBM Corporation, Armonk, NY, USA) was used for all statistical analyses. The R software v.4.2.2 (https://www.r-project.org/, accessed on 6 April 2023) installation package “rms” was used to construct the nomogram. *p* < 0.05 was considered statistically significant.

## 3. Results

### 3.1. Patient and Tumor Characteristics

The study enrolled 76 patients (49 males, 27 females). Table 1 presents the characteristics of the patients. The median follow-up was 86 months (range, 3–125). The NAR score was in the range of 0 to 65 (IQR, 8.43–30.07; median 20.4). Median age was 53 years (range, 19–74 years). Of the cases, BMI < 18.5 kg/m^2^ occurred in 7 cases (9.2%), BMI between 18.5–24.9 kg/m^2^ occurred in 53 cases (69.7%), and BMI ≥ 25 kg/m^2^ occurred in 16 cases (21.1%). Approximately 64.5% of patients had low-lying tumors (within 5 cm of the anal verge). Lymphovascular invasion was present in 15 patients (19.7%), neural invasion in 14 patients (18.4%), and tumor nodules in 16 patients (21.1%). Mucinous adenocarcinoma was observed in 8 patients (10.5%), while 16 patients (21.1%) had poorly differentiated tumors. The distribution of cT stages was as follows: cT2 (4 cases), cT3 (41 cases), and cT4 (31 cases). Lymph-node-positive status was diagnosed in 48 cases (63.2%). After nRT, 55 patients (72.3%) had pathological (yp) T3-4 tumors and 33 patients (43.4%) had ypN0 status. A pCR was achieved in 7 patients (9.2%). Regarding nRT, 36 patients (47.4%) underwent short-course radiotherapy (SCRT), while 40 patients (52.6%) received long-course radiotherapy (LCRT). Neoadjuvant chemotherapy was administered to 63 cases (82.9%). Adjuvant radiotherapy was given to 6 patients (7.9%) and adjuvant chemotherapy to 52 patients (68.4%).

### 3.2. Cutoff Values for Immune Cell Infiltration Characteristics

**Figure 1:**
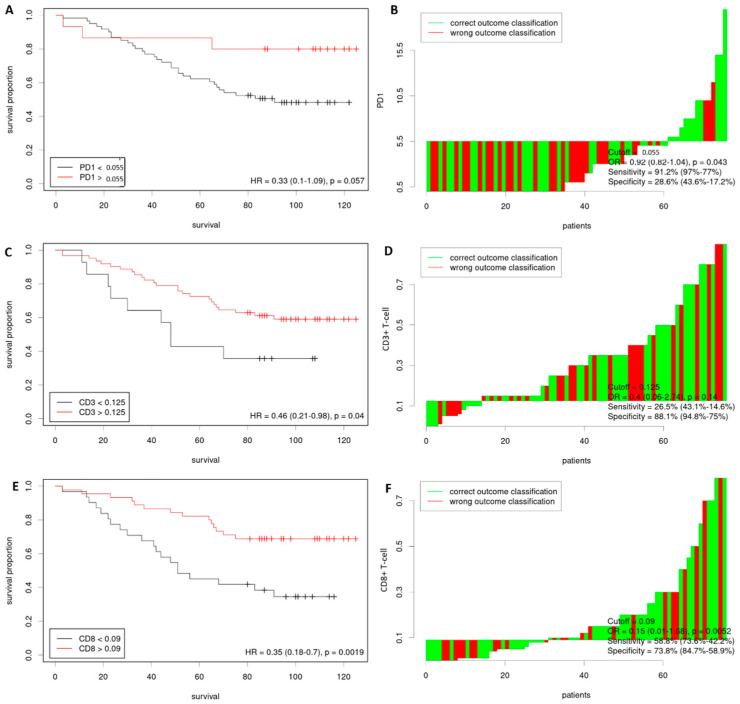
Distribution-based cutoff optimization in LARC patients. Kaplan–Meier curves comparing low vs. high PD-1 (**A**), CD3+ T-cell (**C**), and CD8+ T-cell (**E**). Waterfall plots illustrate the optimal dichotomization of PD1 (**B**), CD3+ T cell (**D**), and CD8+ T cell (**F**) levels. The optimal cutoff was determined based on the occurrence of death events. Abbreviations: LARC, locally advanced rectal cancer; PD1, programmed cell death 1.

Using the Cutoff Finder software via ROC curves, we determined the optimal cutoff value for PD-1 as 5.5% in our cohort (range, 0–20%; median 1%; mean 3.1%). Among the cases, 61 (80.2%) had a low PD-1 expression (<5.5%) and 15 (19.8%) had a high PD-1 expression (≥5.5%). The software also identified an optimal cutoff value of 12.5% for CD3+ T-cells (range, 0%-90%; median 30%; mean 33%). Fourteen cases (18.4%) were classified as having a low CD3+ T-cell count (<12.5%), while 62 cases (81.6%) had a high CD3+ T-cell count (≥12.5%). Additionally, the optimal cutoff value for CD8+ T-cell was 9.0% (range, 0–80%; median 10%; mean 19%). Among the cases, 41 cases (53.9%) had a low CD8+ T-cell count (<9.0%), while 35 cases (46.1%) had a high CD8+ T-cell count (≥9.0%). The results are illustrated in Figure 1 and Table 1.

### 3.3. Independent Prognostic Factors for OS

The results of the univariate and multivariate analyses are presented in Table 2 and Table 3. In the univariate analysis, lymphovascular invasion (HR = 5.148, 95% CI = 2.516–10.533, *p* = 0.000), neural invasion (HR = 2.232, 95% CI = 1.064–4.681, *p* = 0.034), pathologic differentiation (HR = 2.273, 95% CI = 1.086–4.761, *p* = 0.029), ypN stage (HR = 2.460, 95% CI = 1.587–3.814, *p* = 0.000), CD3+ T-cell (HR = 0.459, 95% CI = 0.213–0.987, *p* = 0.046), CD8+ T-cell (HR = 0.354, 95% CI = 0.178–0.703, *p* = 0.003), and NAR score (HR = 1.036, 95% CI = 1.017–1.056, *p* = 0.000) were significantly associated with OS. Additionally, to avoid statistical bias resulting from multicollinearity, NAR, and pathologic stage were not examined within the same model [11]. Multivariate analysis indicated that CD8+ T-cell (HR 0.433, 95% CI 0.198–0.948, *p* = 0.036), lymphovascular invasion (HR 3.375, 95% CI 1.254–9.087, *p* = 0.016), and NAR score (HR 1.028, 95% CI 1.005–1.051, *p* = 0.019) were independent predictors for OS.

### 3.4. The Nomogram for OS

**Figure 2:**
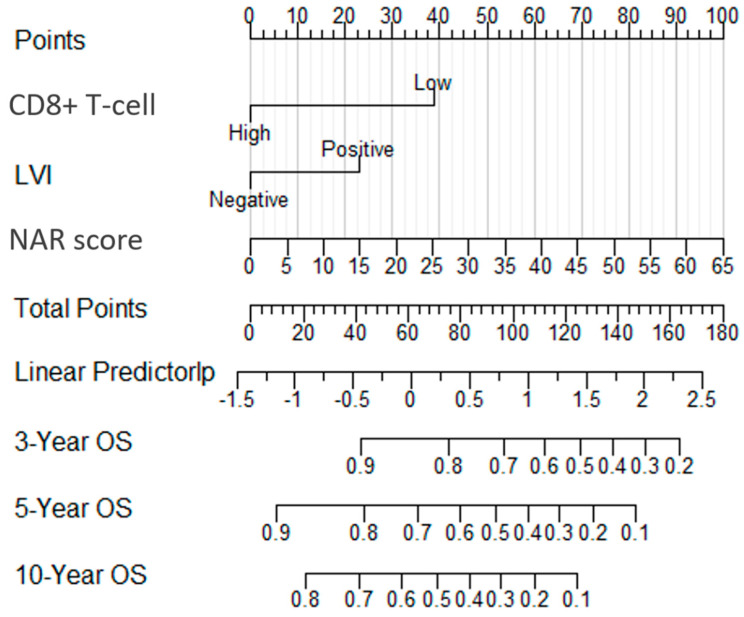
Nomogram incorporating CD8+ T-cell, LVI, and NAR score for predicting risk of death in patients with LARC. The nomogram provides a tool for calculating the probability of death risk by considering the combined effect of these variables. Abbreviations: LVI, Lymphovascular invasion; NAR score, Neoadjuvant rectal score; LARC, Locally advanced rectal cancer; OS, Overall survival.

**Figure 3:**
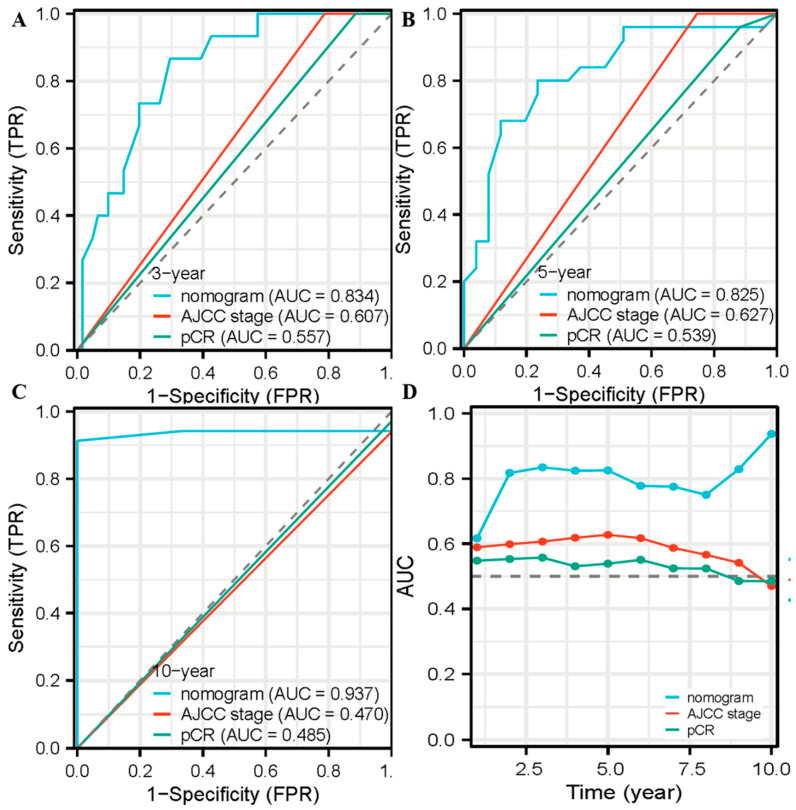
AUC values for the three models predicting OS rates at 3 years (**A**), 5 years (**B**), and 10 years (**C**). (**D**) Time-dependent ROC. Abbreviations: AUC, Area under the curve; OS, Overall survival; ROC, Receiver operating characteristic; AJCC, American Joint Committee on Cancer; pCR, Pathological complete regression.

Subsequently, all independent prognostic factors were incorporated into the nomogram model based on the multivariate analysis (Figure 2). The nomogram scoring was significantly related to TRG grading through Spearman relative analysis (*p* = 0.013). The C-index for OS was 0.739 (95% CI 0.657–0.821), which was higher than that for the AJCC stage (0.587, 95% CI 0.537–0.636), and pCR (0.539, 95% CI 0.495–0.583). Additionally, the C-index for nomogram was superior to that for CD8+ T-cell (0.630, 95% CI 0.549–0.711), lymphovascular invasion (0.606, 95% CI 0.523–0.688) and NAR score (0.696, 95% CI 0.609–0.782). Moreover, the area under the curves (AUCs) were plotted to compare the predictive ability of the nomogram and the other grading systems (Figure 3). The AUCs for OS demonstrated that this nomogram was significantly more predictive than the AJCC stage and pCR at 3-years, 5-years, and 10-years, respectively (3-years OS: nomogram AUC = 0.834, AJCC stage AUC = 0.607, pCR AUC = 0.557, *p* = 0.0289, 0.0039; 5-years OS: nomogram AUC = 0.825, AJCC stage AUC = 0.627, pCR AUC = 0.539, *p* = 0.0001, 8.673836 × 10^−10^; 10-years OS: nomogram AUC = 0.937, AJCC stage AUC = 0.470, pCR AUC = 0.485, *p* = 5.367856 × 10^−58^, 2.100348 × 10^−79^). Over time, the predictions of long-term survival using this model not only remained consistent but also improved in accuracy.

## 4. Discussion

Neoadjuvant chemoradiotherapy followed by TME have been the standard strategies for LARC patients. The response to nRT varies from pCR to minimal effect. Incorporating a novel surrogate short-term end-point is important for assessing the long-term prognosis of LARC treated with nRT. This is the first nomogram combining NAR score and immune cell infiltration characteristics to predict long-term survival to our knowledge. The modified NAR scoring model demonstrates improved accuracy in predicting long-term prognosis compared to the AJCC stage and pCR, making it a more reliable tool over time.

In our cohort, the NAR score (HR 1.028, 95% CI 1.005–1.051, *p* = 0.019) was significantly associated with OS. In our scoring model, CD8+ T-cell (HR 0.433, 95% CI 0.198–0.948, *p* = 0.036), and lymphovascular invasion (HR 3.375, 95% CI 1.254–9.087, *p* = 0.016) were newly added as independent prognostic factors to the modified NAR scoring model. Furthermore, we observed that the new modified-NAR scoring model exhibited a high C-index of 0.739, which was superior to any individual independent factor. Moreover, the predictive value of the nomogram was better than that of the AJCC stage and pCR at 3 years, 5 years, and 10 years (*p* < 0.05). Over time, the predictions of this model for long-term survival not only remained consistent but also improved in accuracy.

DFS and OS are commonly used endpoints in rectal cancer clinical trials, but they require long periods of observation, which slows the pace of research. Previous research has presented varying data regarding the predictive ability of the NAR score in rectal cancer. The score utilizes factors commonly used in clinical work for rectal cancer and requires no additional trial infrastructure, cost, time, or effort. According to the German phase III trial, the NAR score continued to predict DFS independently (low versus high NAR: HR = 4.670, 95% CI 3.106–7.020, *p* < 0.001; low versus intermediate NAR: HR 1.971, 95% CI 1.303–2.98, *p* = 0.001) [16]. However, the baseline staging in that study did not mandate the use of magnetic resonance imaging (MRI), which may have affected the accuracy of the clinical stage. In our cohort, cT and cN stages were assessed via MRI by two radiologists independently to decrease the uncertainty associated with ultrasound staging. Nonconformities were reviewed by a third expert. In a large hospital-based dataset, an unfavorable NAR score (≥14.98) was associated with poor OS (*p* = 0.04) and tumors with perineural invasion had worse NAR scores (*p* = 0.01) [29]. Similarly, among the low, intermediate, and high NAR groups, a significant difference in 5-year OS was observed (93% vs. 88% vs. 75%; *p* < 0.001), and NAR stratification was confirmed to be a significant prognostic factor for 5-year OS (intermediate vs. low, HR = 1.82, 95% CI 1.57–2.10, *p* < 0.001; high vs. low, HR = 3.44, 95% CI 2.94–4.03, *p* < 0.001) [30]. In the NRG-GI002 trial, the mean NAR score was 11.53 for the pembrolizumab arm (95% CI 8.54–14.51) vs. 14.08 for the control arm (95% CI 10.74–17.43) (*p* = 0.26), which was the primary endpoint, although analyses of longer-term results are underway [17]. Therefore, after the long follow-up time, we tried to examine the long-term results here. Furthermore, previous research has shown that the NAR score has a better prognostic value for OS in rectal cancer clinical trials compared to pCR [15]. A nomogram combining the NAR score, TRG, and distance to the anal verge was developed to predict OS and DFS (AUC: 1-year = 0.742, 3-year = 0.749, 5-year = 0.713) [31]. Consistent with previous studies, our study found a significant association between the NAR score and OS (HR 1.028, 95% CI 1.005–1.051, *p* = 0.019). By combining variables related to treatment effects, the NAR score can be used as a surrogate endpoint for short-term efficacy assessments.

Currently, the immune contexture, representing the pre-existing immune parameter, was reported to be associated with cancer prognosis. And, the immunoscore, which combined multiple immune cells, might help to predict the cancer survival [32]. The immunoscore was found to be a predictive factor for neoadjuvant response and long-term prognosis in LARC patients, which was expected to play a role in selecting patients for watch-and-wait strategies [33]. Studies have highlighted the important prognostic value of the immune system in rectal cancer, with tumor-infiltrating lymphocytes (TILs) identified as prognostic factors for favorable oncological outcomes [19,34]. A high density of CD8+ cell (OR 2.69, 95% CI 1.45–4.98, *p* = 0.002) in biopsy samples has been linked with good CRT response, which was considered a good biomarker for predicting outcome after neoadjuvant therapy for rectal cancer [35]. Chemotherapy and radiotherapy have been shown to activate the immune system and synergize with immunotherapy [36]. High density of CD8+ T-cells before CRT has been associated with improved DFS (*p* = 0.0331) [37]. Additionally, the density of CD8+ T-cells increases after chemoradiotherapy (CRT) (*p* < 0.001), and low expression of CD8+ T-cells before and after CRT has been linked to unfavorable DFS (*p* = 0.01) [38]. Moreover, neoadjuvant CRT leads to increased recruitment of CD8+ T-cells within the tumor microenvironment, and high CD8+ T-cell density has been significantly associated with improved DFS after neoadjuvant CRT (*p* = 0.039) [39]. Similarly, in our cohort, the CD8+ T-cell was found to be associated with OS for LARC after nRT (HR 0.433, 95% CI 0.198–0.948, *p* = 0.036).

The incorporation of the NAR score and CD8+ T-cell density into the nomogram resulted in improved predictive accuracy, as evidenced by a high C-index for OS of 0.739. The modified NAR scoring model showed more predictive power than the NAR score (C-index = 0.696) and CD8+ T-cell (C-index = 0.630). Additionally, this was superior to the AJCC stage (C-index = 0.587) and pCR (C-index = 0.539). Furthermore, the modified nomogram exhibited significantly stronger AUC values than the AJCC stage and pCR for 3-year, 5-year, and 10-year OS (*p* < 0.05). Consequently, our nomogram, with its reliance on long-term follow-up, presents a simple and accurate tool for predicting prognosis in LARC patients after nRT. This represents a significant advantage over the AJCC stage and pCR.

Nonetheless, the study has several limitations. Firstly, the results of this single-center retrospective study require further validation. Secondly, although we analyzed immune cell infiltration characteristics such as PD1, CD3+ T-cell, and CD8+ T-cells, some important prognosis-related data were not recorded, such as more immune cells, microsatellite status (MSS), RAS, and BRAF genes. Moreover, the novel modified scoring model would benefit from further validation in prospective studies conducted across multicenter with a large sample size.

## 5. Conclusions

In summary, we successfully developed a modified-NAR scoring model that incorporates immune infiltration characteristics to better predict long-term survival in rectal cancer after nRT. The validation of this model at long-term follow-up demonstrates its accuracy and reliability, which improves over time.

## Figures and Tables

**Table 1:** Characteristics of rectal cancer with neoadjuvant radiotherapy.

Characteristics	Descriptive Analysis	
	n	%
NAR score	MEAN 20.4 ± 16.3	
Age		
<60	56	73.7
≥60	20	26.3
Gender		
Male	49	64.5
Female	27	35.5
BMI (kg/m^2^)		
<18.5	7	9.2
18.5–24.9	53	69.7
≥25	16	21.1
Distance to anal margin (cm)		
<5	49	64.5
≥5	27	35.5
Lymphovascular invasion		
Yes	15	19.7
No	61	80.3
Neural invasion		
Yes	14	18.4
No	62	81.6
Tumor nodules		
Yes	16	21.1
No	60	78.9
Mucinous adenocarcinoma		
Yes	8	10.5
No	68	89.5
Pathologic differentiation		
Poorly	16	21.1
Moderately	60	78.9
cT stage		
2	4	5.3
3	41	53.9
4	31	40.8
cN stage		
0	28	36.8
1	38	50.0
2	10	13.2
ypT stage		
0	4	5.3
2	17	22.4
3	47	61.8
4	8	10.5
ypN stage		
0	33	43.4
1	27	35.5
2	16	21.1
PD-1		
<5.5%	61	80.2
≥5.5%	15	19.8
CD3+ T-cell		
<12.5%	14	18.4
≥12.5%	62	81.6
CD8+ T-cell		
<9.0%	41	53.9
≥9.0%	35	46.1
pCR		
Yes	7	9.2
No	69	90.8
Neoadjuvant radiotherapy		
Short course	36	47.4
Long course	40	52.6
Neoadjuvant chemotherapy		
Yes	63	82.9
No	13	17.1
Adjuvant radiotherapy		
Yes	6	7.9
No	70	92.1
Adjuvant chemotherapy		
Yes	52	68.4
No	24	31.6
Death		
Yes	34	44.7
No	42	55.3

Abbreviations: NAR, Neoadjuvant rectal score; BMI, Body mass index; PD-1, Programmed cell death 1; pCR, Pathological complete regression.

**Table 2:** Variables of LARC Patients Associated with OS According to the Cox Proportional Hazards Regression Model.

Characteristics	Univariable Analysis		
	HR	95% CI	*p*
Age	1.774	0.878–3.587	0.110
Gender	0.629	0.301–1.317	0.219
BMI	1.306	0.685–2.491	0.417
Distance to anal margin (cm)	1.248	0.625–2.494	0.530
Lymphovascular invasion	5.148	2.516–10.533	0.000
Neural invasion	2.232	1.064–4.681	0.034
Tumor nodules	1.844	0.879–3.868	0.106
Mucinous adenocarcinoma	1.990	0.769–5.154	0.156
Pathologic differentiation	2.273	1.086–4.761	0.029
cT stage	0.592	0.332–1.056	0.076
cN stage	1.220	0.742–2.007	0.432
ypT stage	1.136	0.762–1.694	0.531
ypN stage	2.460	1.587–3.814	0.000
PD-1	0.334	0.102–1.095	0.070
CD3+ T-cell	0.459	0.213–0.987	0.046
CD8+ T-cell	0.354	0.178–0.703	0.003
NAR score	1.036	1.017–1.056	0.000
pCR	0.250	0.034–1.832	0.173
Neoadjuvant Radiotherapy	0.582	0.295–1.147	0.118
Neoadjuvant chemotherapy	0.667	0.290–1.532	0.340
Adjuvant radiotherapy	1.080	0.330–3.535	0.898
Adjuvant chemotherapy	0.893	0.435–1.833	0.759

Abbreviations: HR, Hazard ratio; CI, Confidence interval; OS, Overall survival; NAR, Neoadjuvant rectal score; pCR, Pathological complete regression.

**Table 3:** Risk Factors for Survival According to Multivariate Logistic Regression Analyses.

Variable	β Coefficient	HR	95% CI	*p*
Lymphovascular invasion	1.217	3.375	1.254–9.087	0.016
Neural invasion	−0.393	0.675	0.218–2.089	0.496
Pathologic differentiation	0.251	1.286	0.578–2.861	0.538
CD3+ T-cell	−0.298	0.742	0.279–1.976	0.551
CD8+ T-cell	−0.836	0.433	0.198–0.948	0.036
NAR score	0.027	1.028	1.005–1.051	0.019

Abbreviations: HR, Hazard ratio; CI, Confidence interval; NAR, Neoadjuvant rectal score.

## Data Availability

The datasets generated during and/or analyzed during the current study are available from the corresponding author on reasonable request.

## References

[1] Siegel R.L., Miller K.D., Wagle N.S., Jemal A. (2023). Cancer statistics, 2023. CA Cancer J. Clin..

[2] Sauer R., Liersch T., Merkel S., Fietkau R., Hohenberger W., Hess C., Becker H., Raab H.R., Villanueva M.T., Witzigmann H. (2012). Preoperative versus postoperative chemoradiotherapy for locally advanced rectal cancer: Results of the German CAO/ARO/AIO-94 randomized phase III trial after a median follow-up of 11 years. J. Clin. Oncol..

[3] Luo D., Yang Y., Zhang R., Li Q., Li X. (2023). Effect of interval between neoadjuvant chemoradiotherapy and surgery on oncological outcomes in poor responders with locally advanced rectal cancer: A retrospective cohort study. Int. J. Surg..

[4] Sauer R., Becker H., Hohenberger W., Rodel C., Wittekind C., Fietkau R., Martus P., Tschmelitsch J., Hager E., Hess C.F. (2004). Preoperative versus postoperative chemoradiotherapy for rectal cancer. N. Engl. J. Med..

[5] Buyse M., Molenberghs G., Paoletti X., Oba K., Alonso A., Van der Elst W., Burzykowski T. (2016). Statistical evaluation of surrogate endpoints with examples from cancer clinical trials. Biom. J..

[6] Quah H.M., Chou J.F., Gonen M., Shia J., Schrag D., Saltz L.B., Goodman K.A., Minsky B.D., Wong W.D., Weiser M.R. (2008). Pathologic stage is most prognostic of disease-free survival in locally advanced rectal cancer patients after preoperative chemoradiation. Cancer.

[7] Fokas E., Liersch T., Fietkau R., Hohenberger W., Hess C., Becker H., Sauer R., Wittekind C., Rodel C. (2015). Downstage migration after neoadjuvant chemoradiotherapy for rectal cancer: The reverse of the Will Rogers phenomenon?. Cancer.

[8] Fokas E., Liersch T., Fietkau R., Hohenberger W., Beissbarth T., Hess C., Becker H., Ghadimi M., Mrak K., Merkel S. (2014). Tumor regression grading after preoperative chemoradiotherapy for locally advanced rectal carcinoma revisited: Updated results of the CAO/ARO/AIO-94 trial. J. Clin. Oncol..

[9] Liu Y., Zhang F.J., Zhao X.X., Yang Y., Liang C.Y., Feng L.L., Wan X.B., Ding Y., Zhang Y.W. (2021). Development of a Joint Prediction Model Based on Both the Radiomics and Clinical Factors for Predicting the Tumor Response to Neoadjuvant Chemoradiotherapy in Patients with Locally Advanced Rectal Cancer. Cancer Manag. Res..

[10] Germani P., Di Candido F., Léonard D., Cuicchi D., Elmore U., Allaix M.E., Barbieri V.P., D’Allens L., Faes S., Milani M. (2021). Contemporary snapshot of tumor regression grade (TRG) distribution in locally advanced rectal cancer: A cross sectional multicentric experience. Updates Surg..

[11] Erlandsson J., Lörinc E., Ahlberg M., Pettersson D., Holm T., Glimelius B., Martling A. (2019). Tumour regression after radiotherapy for rectal cancer—Results from the randomised Stockholm III trial. Radiother. Oncol..

[12] Park I.J., You Y.N., Agarwal A., Skibber J.M., Rodriguez-Bigas M.A., Eng C., Feig B.W., Das P., Krishnan S., Crane C.H. (2012). Neoadjuvant treatment response as an early response indicator for patients with rectal cancer. J. Clin. Oncol..

[13] Glynne-Jones R., Hughes R. (2012). Critical appraisal of the ‘wait and see’ approach in rectal cancer for clinical complete responders after chemoradiation. Br. J. Surg..

[14] Valentini V., van Stiphout R.G., Lammering G., Gambacorta M.A., Barba M.C., Bebenek M., Bonnetain F., Bosset J.F., Bujko K., Cionini L. (2011). Nomograms for predicting local recurrence, distant metastases, and overall survival for patients with locally advanced rectal cancer on the basis of European randomized clinical trials. J. Clin. Oncol..

[15] George T.J., Allegra C.J., Yothers G. (2015). Neoadjuvant Rectal (NAR) Score: A New Surrogate Endpoint in Rectal Cancer Clinical Trials. Curr. Color. Cancer Rep..

[16] Fokas E., Fietkau R., Hartmann A., Hohenberger W., Grutzmann R., Ghadimi M., Liersch T., Strobel P., Grabenbauer G.G., Graeven U. (2018). Neoadjuvant rectal score as individual-level surrogate for disease-free survival in rectal cancer in the CAO/ARO/AIO-04 randomized phase III trial. Ann. Oncol..

[17] Rahma O.E., Yothers G., Hong T.S., Russell M.M., You Y.N., Parker W., Jacobs S.A., Colangelo L.H., Lucas P.C., Gollub M.J. (2021). Use of Total Neoadjuvant Therapy for Locally Advanced Rectal Cancer: Initial Results From the Pembrolizumab Arm of a Phase 2 Randomized Clinical Trial. JAMA Oncol..

[18] Drake C.G., Lipson E.J., Brahmer J.R. (2014). Breathing new life into immunotherapy: Review of melanoma, lung and kidney cancer. Nat. Rev. Clin. Oncol..

[19] Otegbeye E.E., Mitchem J.B., Park H., Chaudhuri A.A., Kim H., Mutch M.G., Ciorba M.A. (2021). Immunity, immunotherapy, and rectal cancer: A clinical and translational science review. Transl. Res..

[20] Weichselbaum R.R., Liang H., Deng L., Fu Y.X. (2017). Radiotherapy and immunotherapy: A beneficial liaison?. Nat. Rev. Clin. Oncol..

[21] Wang Y., Shen L., Wan J., Zhang H., Wu R., Wang J., Wang Y., Xu Y., Cai S., Zhang Z. (2022). Neoadjuvant chemoradiotherapy combined with immunotherapy for locally advanced rectal cancer: A new era for anal preservation. Front. Immunol..

[22] Lin Z., Cai M., Zhang P., Li G., Liu T., Li X., Cai K., Nie X., Wang J., Liu J. (2021). Phase II, single-arm trial of preoperative short-course radiotherapy followed by chemotherapy and camrelizumab in locally advanced rectal cancer. J. Immunother. Cancer.

[23] Heo J., Oh Y.T., Noh O.K., Chun M., Park J.E., Cho S.R. (2016). Nodal tumor response according to the count of peripheral blood lymphocyte subpopulations during preoperative chemoradiotherapy in locally advanced rectal cancer. Radiat. Oncol. J..

[24] Fang P., Jiang W., Davuluri R., Xu C., Krishnan S., Mohan R., Koong A.C., Hsu C.C., Lin S.H. (2018). High lymphocyte count during neoadjuvant chemoradiotherapy is associated with improved pathologic complete response in esophageal cancer. Radiother. Oncol..

[25] Denkert C., von Minckwitz G., Brase J.C., Sinn B.V., Gade S., Kronenwett R., Pfitzner B.M., Salat C., Loi S., Schmitt W.D. (2015). Tumor-infiltrating lymphocytes and response to neoadjuvant chemotherapy with or without carboplatin in human epidermal growth factor receptor 2-positive and triple-negative primary breast cancers. J. Clin. Oncol..

[26] Chen C.C., Wu M.L., Huang K.C., Huang I.P., Chung Y.L. (2020). The Effects of Neoadjuvant Treatment on the Tumor Microenvironment in Rectal Cancer: Implications for Immune Activation and Therapy Response. Clin. Color. Cancer.

[27] Mandard A.M., Dalibard F., Mandard J.C., Marnay J., Henry-Amar M., Petiot J.F., Roussel A., Jacob J.H., Segol P., Samama G. (1994). Pathologic assessment of tumor regression after preoperative chemoradiotherapy of esophageal carcinoma. Clinicopathologic correlations. Cancer.

[28] Budczies J., Klauschen F., Sinn B.V., Gyorffy B., Schmitt W.D., Darb-Esfahani S., Denkert C. (2012). Cutoff Finder: A comprehensive and straightforward Web application enabling rapid biomarker cutoff optimization. PLoS ONE.

[29] Shah S., Asawa P., Abel S., Wegner R.E. (2022). Validation of the Neoadjuvant Rectal Cancer (NAR) Score for Prognostication Following Total Neoadjuvant Therapy (TNT) for Locally Advanced Rectal Cancer. J. Gastrointest. Cancer.

[30] Naffouje S.A., Manguso N., Imanirad I., Sahin I.H., Xie H., Hoffe S., Frakes J., Sanchez J., Dessureault S., Felder S. (2022). Neoadjuvant rectal score is prognostic for survival: A population-based propensity-matched analysis. J. Surg. Oncol..

[31] Wang G., Tang Z., Ye J., Tang H., Yao K., Zeng Q., Yang Y., Fu M., Luo L., Shen Q. (2023). Development and validation of neoadjuvant rectal score-based signature nomograms to predict overall survival and disease-free survival in locally advanced rectal cancer: A retrospective, double center, cohort study. Int. J. Clin. Oncol..

[32] Bruni D., Angell H.K., Galon J. (2020). The immune contexture and Immunoscore in cancer prognosis and therapeutic efficacy. Nat. Rev. Cancer.

[33] El Sissy C., Kirilovsky A., Van den Eynde M., Muşină A.M., Anitei M.G., Romero A., Marliot F., Junca A., Doyen J., Mlecnik B. (2020). A Diagnostic Biopsy-Adapted Immunoscore Predicts Response to Neoadjuvant Treatment and Selects Patients with Rectal Cancer Eligible for a Watch-and-Wait Strategy. Clin. Cancer Res..

[34] Shinto E., Hase K., Hashiguchi Y., Sekizawa A., Ueno H., Shikina A., Kajiwara Y., Kobayashi H., Ishiguro M., Yamamoto J. (2014). CD8+ and FOXP3+ tumor-infiltrating T cells before and after chemoradiotherapy for rectal cancer. Ann. Surg. Oncol..

[35] Sano S., Akiyoshi T., Yamamoto N., Hiyoshi Y., Mukai T., Yamaguchi T., Nagasaki T., Taketomi A., Fukunaga Y., Kawachi H. (2023). Intratumoral Budding and CD8-Positive T-cell Density in Pretreatment Biopsies as a Predictor of Response to Neoadjuvant Chemoradiotherapy in Advanced Rectal Cancer. Clin. Color. Cancer.

[36] McLaughlin M., Patin E.C., Pedersen M., Wilkins A., Dillon M.T., Melcher A.A., Harrington K.J. (2020). Inflammatory microenvironment remodelling by tumour cells after radiotherapy. Nat. Rev. Cancer.

[37] Ogura A., Akiyoshi T., Yamamoto N., Kawachi H., Ishikawa Y., Mori S., Oba K., Nagino M., Fukunaga Y., Ueno M. (2018). Pattern of programmed cell death-ligand 1 expression and CD8-positive T-cell infiltration before and after chemoradiotherapy in rectal cancer. Eur. J. Cancer.

[38] Lim Y.J., Koh J., Kim S., Jeon S.R., Chie E.K., Kim K., Kang G.H., Han S.W., Kim T.Y., Jeong S.Y. (2017). Chemoradiation-Induced Alteration of Programmed Death-Ligand 1 and CD8(+) Tumor-Infiltrating Lymphocytes Identified Patients With Poor Prognosis in Rectal Cancer: A Matched Comparison Analysis. Int. J. Radiat. Oncol. Biol. Phys..

[39] Chen T.W., Huang K.C., Chiang S.F., Chen W.T., Ke T.W., Chao K.S.C. (2019). Prognostic relevance of programmed cell death-ligand 1 expression and CD8+ TILs in rectal cancer patients before and after neoadjuvant chemoradiotherapy. J. Cancer Res. Clin. Oncol..

